# Real-World Treatment Patterns, Sequencing, and Outcomes in Patients with Locally Advanced or Metastatic Urothelial Carcinoma Receiving Avelumab First-Line Maintenance in the United States

**DOI:** 10.3390/curroncol31090420

**Published:** 2024-09-21

**Authors:** Helen H. Moon, Mairead Kearney, Seyed Hamidreza Mahmoudpour, Chiemeka Ike, Valerie Morris, Andrew Rava, Sonia Kim, Haiyan Sun, Marley Boyd, Gabriel Gomez Rey

**Affiliations:** 1 Kaiser Permanente Southern California, Riverside, CA 92505, USA; 2 Merck Healthcare KGaA, 64293 Darmstadt, Germany; mairead.kearney@merckgroup.com (M.K.); hamid.mahmoudpour@merckgroup.com (S.H.M.); 3 EMD Serono, Inc., Rockland, MA 02370, USA; chiemeka.ike@merckgroup.com (C.I.); valerie.morris@merckgroup.com (V.M.); 4 Genesis Research Group, Hoboken, NJ 07030, USA; andrew@genesisrg.com (A.R.); skim@genesisrg.com (S.K.); haiyan@genesisrg.com (H.S.); marley.boyd@genesisrg.com (M.B.); ggomezrey@hotmail.com (G.G.R.)

**Keywords:** urothelial carcinoma, real world, treatment sequencing, clinical outcomes, survival, platinum-based chemotherapy, avelumab, enfortumab vedotin, community oncology

## Abstract

For patients with locally advanced/metastatic urothelial carcinoma (la/mUC), first-line (1L) treatment with platinum-based chemotherapy (PBC) followed by avelumab 1L maintenance (1LM) is a recommended therapy per treatment guidelines in patients without disease progression. However, contemporary real-world (rw) data among patients receiving this treatment are necessary to understand clinical outcomes and optimal treatment sequencing. This retrospective cohort study analyzed rw treatment patterns and clinical outcomes, including overall survival (rwOS) and progression-free survival (rwPFS), in patients with la/mUC receiving avelumab 1LM. From the Flatiron Health database, 214 patients who received avelumab 1LM following 1L PBC were included. From the start of avelumab 1LM, median rwOS was 23.8 months (95% CI: 18.2—not estimable [NE]) and median rwPFS was 5.1 months (95% CI: 4.1–7.0). A total of 96 patients received second-line (2L) therapy, with 53 receiving enfortumab vedotin (EV). From the start of 2L EV, median rwOS was 11.2 months (95% CI: 6.8—NE) and median rwPFS was 4.9 months (95% CI: 3.9–8.8). Treatment patterns and clinical outcomes in this study align with guidelines and outcomes observed in the JAVELIN Bladder 100 and EV-301 clinical trials and other rw studies, supporting the use of 1L PBC followed by avelumab 1LM and 2L EV for eligible patients.

## 1. Introduction

Bladder cancer, which is primarily diagnosed as urothelial carcinoma (UC), accounted for 70,306 new cases of cancer and 16,682 deaths in the United States (US) in 2020 [1]. In 2024, an estimated 83,190 new cases and 16,840 deaths are projected [2]. Advanced UC (aUC), which comprises locally advanced (la) and metastatic (m) disease, accounts for approximately 12% of bladder cancer cases in the US [3]. The 5-year relative survival rates for patients diagnosed with advanced disease are poor: 39.2% for regional stage disease and 8.3% for distant stage disease [3].

The NCCN Clinical Practice Guidelines in Oncology (NCCN Guidelines^®^) recommend treatment with 1L platinum-based chemotherapy (PBC), either cisplatin plus gemcitabine or carboplatin plus gemcitabine, followed by avelumab 1L maintenance (1LM; in patients without disease progression after 1L PBC), as a Category 1 ‘other recommended’ regimen [4]. This recommendation was based on results of the pivotal JAVELIN Bladder 100 trial (JB100; NCT02603432), which found that avelumab 1LM plus best supportive care (BSC) improved overall survival (OS) and progression-free survival (PFS) compared with BSC alone, with a median OS of 21.4 vs. 14.3 months and a median PFS of 3.7 vs. 2.0 months, in patients without disease progression following 1L PBC [5]. After ≥2 years of follow-up in all patients (data cutoff: June 4, 2021), median OS from the start of avelumab 1LM plus BSC vs. BSC alone was 23.8 vs. 15.0 months, and median PFS was 5.5 vs. 2.1 months [6]. Cisplatin-based therapy is the preferred choice of 1L PBC, with carboplatin-based therapy used as an alternative for the 30–50% of patients not eligible for cisplatin [7,8]. Ineligibility criteria for any platinum-based therapy include the following: (1) Eastern Cooperative Oncology Group performance status (ECOG PS) of ≥3, (2) creatinine clearance of <30 mL/min, (3) grade ≥2 peripheral neuropathy, (4) New York Heart Association heart failure class of >3, or (5) ECOG PS of 2 and creatinine clearance of <30 mL/min [9]. After 1L PBC and avelumab 1LM or 2L immuno-oncology (IO), NCCN-preferred therapies include EV monotherapy [10] and erdafitinib [11,12], with other recommended therapies including the antibody-drug conjugate (ADC) therapies sacituzumab govitecan [13] and trastuzumab deruxtecan [14] as well as various chemotherapy regimens [4].

As the treatment landscape has evolved to include ADCs and targeted therapies more broadly, contemporary real-world data among patients receiving 1L PBC followed by avelumab 1LM are needed to understand clinical outcomes and optimal treatment sequencing. This study aimed to describe baseline demographic and clinical characteristics, real-world treatment patterns, and clinical outcomes of patients with la/mUC in the US who received avelumab 1LM following treatment with 1L PBC. Characterizing treatment sequencing patterns and outcomes following disease progression on avelumab 1LM in a rapidly evolving therapeutic landscape was a key objective of this analysis. This study examined the clinical outcomes in patients treated with avelumab 1LM and those treated with contemporary 2L therapies that may not have been approved at the time of JB100.

## 2. Methods

### 2.1. Data Source and Study Design

This noninterventional, retrospective, cohort study analyzed patients with la/mUC in the US using data from Flatiron Health’s electronic health records (EHR) spotlight. The Flatiron Health database is a longitudinal, demographically and geographically diverse database composed of de-identified, patient-level, structured and unstructured data originating from >280 community cancer centers and 8 major academic cancer centers across the US, accounting for >3 million patient records (75% from community practices, 25% from academic cancer centers) [15,16,17]. This study used data from the Flatiron Health Advanced Urothelial Carcinoma database, which is a subset of the Flatiron Health database containing patients diagnosed with aUC.

**Figure 1:**
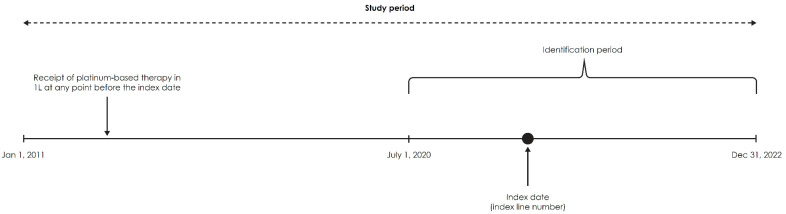
Study design. Abbreviations: 1L, first line.

The study period was from 1 January 2011 through 31 December 2022. The index date was defined as the initiation date of avelumab 1LM in the identification period (1 July 2020 through 31 December 2022). For time-to-event outcomes in other lines of therapy (LOTs) such as 2L, participants were followed up from the index line of interest (2L) (Figure 1). The institutional review board (IRB) approval of this observational study with secondary data use from an existing EHR database was covered by the Flatiron Health parent protocol. This study was exempt from additional IRB approval because it was retrospective, was noninterventional, and used anonymized data provided by Flatiron Health. The study was conducted in accordance with legal and regulatory requirements, as well as with scientific purpose, value, and rigor, and followed generally accepted research practices for retrospective observational studies, described in Guidelines for Good Pharmacoepidemiology Practices (GPP) issued by the International Society for Pharmacoepidemiology (ISPE) [18], Good Practices for Outcomes Research issued by the International Society for Pharmacoeconomics and Outcomes Research (ISPOR) [19], and the GRACE (Good Research for Comparative Effectiveness) Principles [20].

### 2.2. Study Population

The patient inclusion criteria for the study were as follows: (1) diagnosed with UC (*International Classification of Diseases*, *Ninth Revision* [ICD-9] codes 188x, 189.1, 189.2, or 189.3 or ICD-10 codes C65x, C66x, C67x, or C68.0); (2) ≥2 documented clinic visits in the Flatiron Health database, occurring on different days, on or after 1 January 2011; (3) pathology consistent with transitional cell (urothelial) carcinoma; (4) diagnosed with stage IV UC or node-positive UC on or after 1 January 2011, or diagnosed with early-stage UC and subsequently developed advanced disease on or after 1 January 2011; (5) advanced diagnosis date on or after 1 January 2019; (6) evidence of treatment with PBC (cisplatin, carboplatin, oxaliplatin), alone or in combination with any other therapy in 1L for la/mUC, as defined per Flatiron Health’s LOT business rules; and (7) evidence of treatment with avelumab in any line for la/mUC, alone or in combination with any other drug(s) on or after 1 July 2020. The following additional inclusion criteria were used to define avelumab 1LM: (8) received avelumab within 90 days after 1L PBC discontinuation; (9) did not have >1 LOT before initiating avelumab 1LM; and (10) did not have disease progression (from Flatiron Health’s progression table) within 8–14 weeks after last administration/order of PBC in 1L. Patient exclusion criteria for the study were as follows: (1) pediatric patients (age < 18 years) at index date; (2) lacking relevant unstructured documents in the Flatiron Health database for review by the abstract team; or (3) received treatment with a clinical study drug alone or in combination with any other drug(s) in 1L, as defined per Flatiron Health’s LOT business rules.

### 2.3. Study Measures

Patient demographics and clinical characteristics were described from the date of la/mUC diagnosis or index date. Patterns and sequencing were described for 1L, 1LM, 2L, and third-line (3L) treatments. LOTs were defined based on the oncologist-defined LOT rules built into the Flatiron Health database. The rules are as follows: (1) the first eligible therapy was received after or up to 14 days before the index date and after the start of the patient’s structured activity; (2) receipt of the first eligible therapy plus other eligible therapies was given within 28 days; (3) across all drugs that make up a LOT, a gap of >120 days between any 2 sequential receipts of therapy would advance the LOT number; (4) for the most recent LOT, the end date was defined as the last date of patient-level structured activity or death, whichever occurred first; and (5) for earlier LOTs, the end date was the day before the start date of the next LOT. Avelumab’s use as 1LM was defined using the algorithm defined in inclusion criteria 8–10. Treatment sequences for each patient were assessed from 1L to 1LM (pre-index) to 2L (post-index) to 3L (post-index), based on the treatment groups within each line. Sequences were reported as a categorical variable (n, %).

Clinical outcomes included best response to treatment, objective response rate (ORR), real-world OS (rwOS), real-world PFS (rwPFS), time to treatment discontinuation (TTD), and time to next treatment (TTNT). Among patients with several recorded responses to treatment, the best recorded response was considered. Responses were defined by clinicians as a categorical response to therapy. Best response was reported as real-world complete response (rwCR), real-world partial response (rwPR), or real-world stable disease (rwSD). ORR was calculated as the proportion of patients with a best response of either rwCR or rwPR out of the total number of patients with a recorded response assessment. rwOS and rwPFS were assessed from the start of avelumab 1LM and the start of 2L EV. rwOS was defined as the interval (months) between initiation of the relevant index treatment until date of death, as documented by the Flatiron Health database. If patients did not have evidence of death, they were censored at last contact in the Flatiron Health database or at study cutoff. rwPFS was defined as the interval (months) between initiation of the relevant index treatment until evidence of first progression or death. If no evidence of progression existed, the interval ended at date of death (any cause) as documented by the Flatiron Health database. If patients did not have evidence of progression or death, they were censored at last contact in the Flatiron Health database, study cutoff, or initiation of a new anticancer therapy leading to a change in LOT. TTD was defined as the time (months) between LOT start date and LOT end date (date of treatment discontinuation or death due to any cause, whichever occurred first). TTNT was defined as the time (months) between LOT start date and next LOT start date or death due to any cause, whichever occurred first.

### 2.4. Statistical Analysis

This study was designed as a descriptive, noncomparative analysis. Continuous variables were summarized using means, standard deviations (Std Devs), medians, 25th percentiles (Q1), and 75th percentiles (Q3). Categorical variables were summarized using frequencies and percentages. Missing data (unknown/not documented) were considered a separate category in all analyses and described using frequency counts and percentages. Treatment sequences by LOT over time were illustrated using a Sankey flow diagram. Kaplan–Meier curves were used to estimate rwOS and rwPFS from initiation of avelumab 1LM and 2L EV therapy. For rwOS, rwPFS, TTD, and TTNT, medians with 95% CIs were reported. Statistical analyses were conducted using SAS, version 9.4 (SAS Institute Inc., Cary, NC, USA).

## 3. Results

### 3.1. Baseline Demographics and Clinical Characteristics

**Figure 2:**
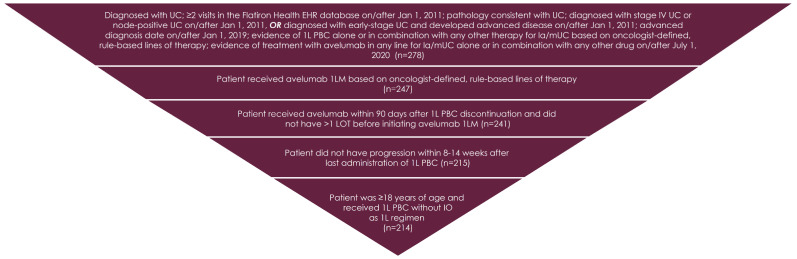
Patient population. Abbreviations: 1L, first line; 1LM, first-line maintenance; EHR, electronic healthcare record; IO, immuno-oncology; la/mUC, locally advanced/metastatic urothelial carcinoma; LOT, line of therapy; PBC, platinum-based chemotherapy; UC, urothelial carcinoma.

After applying the inclusion criteria implemented by Flatiron Health, 278 patients with la/mUC were selected, 214 of whom met the inclusion criteria for this study, which were established to accurately identify patients who were eligible for and received avelumab 1LM (Figure 2). Mean age was 69.0 years (Std Dev: 9.2). Most patients were male (76.6%) and White (66.4%), with an ECOG PS of 0 (32.2%) or 1 (44.9%) (Table 1). Similar proportions were seen for the body mass index (BMI) categories of normal BMI (32.2%), overweight BMI (27.6%), and obese BMI (28.0%). The majority (73.8%) of primary tumors occurred in the bladder. The most common sites of metastases were distant lymph nodes (57.0%), bone (29.4%), and lungs (27.1%).

### 3.2. Treatment Patterns/Sequencing

The current study examined the sequence of treatments from 1L PBC through avelumab 1LM and subsequent treatments. Among the 214 patients who received 1L PBC followed by avelumab 1LM, 115 (53.7%) received cisplatin-based therapy and 99 (46.3%) received carboplatin-based therapy (Table 1). The most prevalent cisplatin-based regimen was cisplatin plus gemcitabine (n = 101, 87.8%), and the most prevalent carboplatin-based regimen was carboplatin plus gemcitabine (n = 98, 99.0%). Most patients had a best response to 1L PBC of rwPR (n = 159, 78.7%), with 14 patients (6.9%) having a rwCR and 29 patients (14.4%) having rwSD.

**Figure 3:**
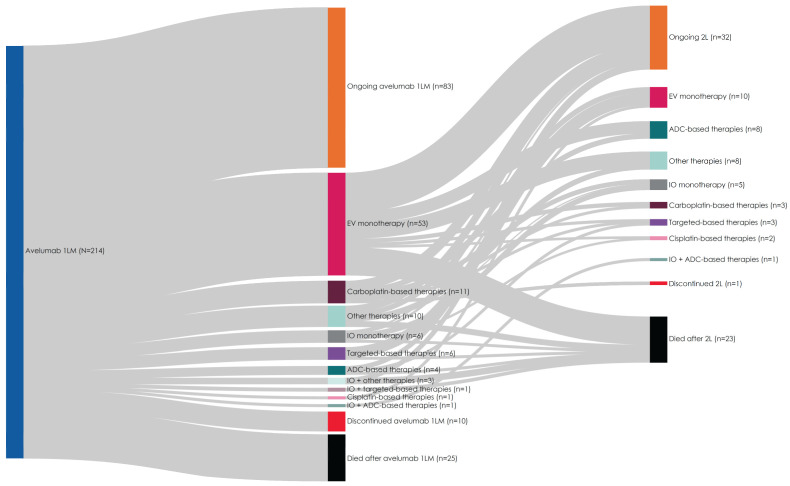
Treatment sequencing from 1LM to 2L to 3L treatment regimens during the observation period. Abbreviations: 1LM, first-line maintenance; 2L, second line; 3L, third line; ADC, antibody–drug conjugate; EV, enfortumab vedotin; IO, immuno-oncology.

Most patients (83.6%) either remained on avelumab 1LM through the study period end (n = 83, 38.8%) or received 2L treatment (n = 96, 44.9%) (Figure 3). Of patients receiving 2L treatment, the most common was EV monotherapy (n = 53, 55.2%). Additional 2L treatments categories included carboplatin-based therapies (n = 11), IO monotherapy (n = 6), target-based therapies (n = 6), other ADC-based therapies (n = 4), IO plus other therapies (n = 3), IO plus targeted-based therapies (n = 1), cisplatin-based therapies (n = 1), IO plus ADC-based therapies (n = 1), and other therapies (docetaxel [n = 1]; docetaxel plus leuprolide [n = 1]; fluorouracil plus mitomycin [n = 1]; gemcitabine plus paclitaxel [n = 2]; letrozole [n = 1]; methotrexate [n = 1]; paclitaxel [n = 2]; topotecan [n = 1]).

Of 96 patients who received 2L treatment, 32 (33.3%) had ongoing 2L treatment at data cutoff, while 40 (41.7%) received 3L treatment. EV monotherapy (n = 10, 25.0%) was the most common 3L treatment, with half of these patients (n = 5) receiving 2L targeted therapy with erdafitinib. Other 3L therapies included other ADCs (n = 8), IO monotherapy (n = 5), carboplatin-based therapies (n = 3), targeted therapies (n = 3), cisplatin-based therapies (n = 2), IO plus ADC (n = 1), and other therapies (n = 8).

### 3.3. Clinical Outcomes

**Figure 4:**
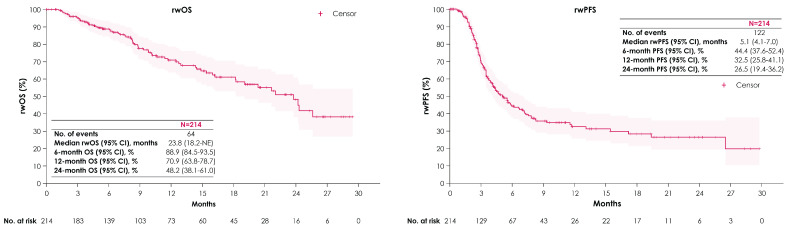
rwOS and rwPFS from avelumab 1LM initiation. Abbreviations: 1LM, first-line maintenance; NE, not estimable; rwOS, real-world overall survival; rwPFS, real-world progression-free survival.

The median duration of avelumab 1LM treatment was 3.9 months (Q1, Q3: 1.9, 7.2), with a median follow-up of 8.7 months (Q1, Q3: 4.5, 15.7) (Table 2). Among the 171 patients with a recorded response to avelumab 1LM, the best response included 16 (9.4%) with a rwCR, 44 (25.7%) with a rwPR, and 44 (25.7%) with rwSD. ORR was 35.1% (95% CI: 28.0–42.7). Among the 214 patients, median rwOS and rwPFS from the start of avelumab 1LM were 23.8 months (95% CI: 18.2—not estimable [NE]) and 5.1 months (95% CI: 4.1–7.0), respectively (Figure 4). Median TTD from avelumab 1LM initiation was 4.9 months (95% CI: 4.2–6.5). Median TTNT from avelumab 1LM initiation was 7.0 months (95% CI: 5.6–8.6).

**Figure 5:**
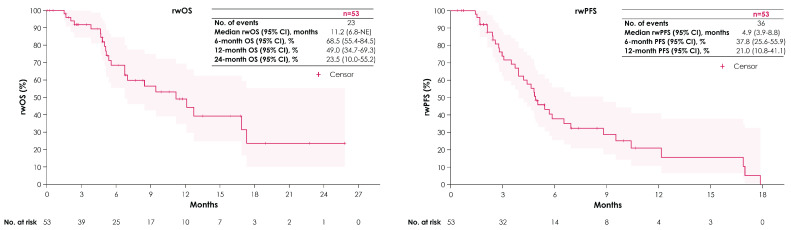
rwOS and rwPFS from 2L EV initiation. Abbreviations: 2L, second line; EV, enfortumab vedotin; NE, not estimable; rwOS, real-world overall survival; rwPFS, real-world progression-free survival.

Of the 53 patients who received 2L EV after avelumab 1LM, the median duration of 2L EV treatment was 4.2 months (Q1, Q3: 1.6, 6.3) (Table 2). Median rwOS and rwPFS from 2L EV initiation were 11.2 months (95% CI: 6.8—NE) and 4.9 months (95% CI: 3.9–8.8), respectively (Figure 5). Median TTD from 2L EV initiation was 4.7 months (95% CI: 4.2–8.5). Median TTNT from 2L EV initiation was 5.8 months (95% CI: 5.2–9.5).

## 4. Discussion

This study provides real-world data on treatment patterns and clinical outcomes in US patients with aUC and offers insight into the clinical benefit of treatments in a patient population not well represented in clinical trials. Among this group of 214 patients that received avelumab 1LM after PBC, most (84%, n = 179) either received 2L therapy or remained on 1LM through the end of the study period, with 19% (n = 40) receiving 3L therapy. EV was the most common 2L treatment (n = 53, 55%), as reported in similar real-world studies [21,22] and in agreement with NCCN Guidelines as a preferred therapy following avelumab 1LM [4].

Although PBC combination was the sole established 1L therapy for aUC for decades, treatment options are rapidly expanding, reflected in the most recent NCCN Guidelines [4]. New frontline options include the Category 1, ‘preferred’ treatment of pembrolizumab plus EV based on the results of the EV-302 trial (NCT04223856) [23] and the Category 1, ‘other recommended’ treatment of nivolumab, gemcitabine, and cisplatin followed by nivolumab maintenance based on the results of the CheckMate-901 trial substudy (NCT03036098) [24]. Considering these recent additions, this study highlights the role the JAVELIN paradigm of PBC followed by avelumab 1LM continues to play. Despite the emergence of multiple treatment options over the past few years, it is important to remember that the vast majority of patients with aUC are still not cured. There remains an opportunity to further improve the survival outcomes in patients by ensuring that the frontline treatment strategy chosen considers subsequent LOTs and is supported by evidence-based recommendations.

It is often suspected there is a discordance between clinical trials and real-world outcomes in part due to strict eligibility criteria that limit the external generalizability or validity of the study results. It is widely believed patients in clinical trials are usually healthier with superior performance status, and real-world data provide an opportunity to validate the outcomes seen in clinical trials. This was seen in our study; compared with patients receiving avelumab 1LM in the JB100 trial [6], patients in this study were slightly older (68.0 vs. 70.0 years) and had poorer performance status—a higher proportion of patients in JB100 had a baseline ECOG PS of 0 (n = 213, 60.9%) vs. in this study (n = 69, 32.2%). Meanwhile, this study also included patients with an ECOG PS of ≥2 (n = 21, 9.8%), who would not have met the strict eligibility criteria of ECOG PS of 0 or 1 required by the JB100 trial to receive treatment. Additionally, the best response to 1L PBC differed between the studies. In the JB100 trial, 25.7% (n = 90) had a CR, 46.6% (n = 163) had a PR, and 27.7% (n = 97) had SD. In contrast, among the 202 patients in this study with a recorded response to 1L PBC, most had rwPR (n = 159, 78.7%), with fewer patients having rwCR (n = 14, 6.9%) or rwSD (n = 29, 14.4%). Differences in study design (clinical trial vs. real-world study), study populations (global vs. US only), and clinicians’ determination and interpretation of response, which was verified by blinded centralized review in JB100, may have contributed to these differences in response between the two studies.

It is satisfying to see that despite the differences in these study populations, outcomes from avelumab 1LM initiation in this study align with those observed in JB100 [6]. Although this study had a shorter median treatment duration vs. JB100, possibly due to shorter follow-up (8.7 vs. 38.0 months) and differences in the characteristics of study populations, OS and PFS were comparable [6]. Median rwOS was 23.8 months (95% CI: 18.2—NE) compared with a median OS in JB100 of 23.8 months (95% CI: 19.9–28.8), and rwPFS was 5.1 months (95% CI: 4.1–7.0) compared with a median PFS in JB100 of 5.5 months (95% CI: 4.2–7.2) [6]. These results are further supported by other large real-world studies with longer follow-up times from the US (PATRIOT-II) [25], Italy (READY CUP) [26], and France (AVENANCE) [27]. Comparable values were reported for median rwOS in PATRIOT-II (24.4 months), READY CUP (26.2 months), and AVENANCE (21.1 months) as well as median rwPFS in PATRIOT-II (5.4 months) and READY CUP (7.6 months) [25,26,27], further validating the JB100 trial results and supporting the real-world efficacy of avelumab 1LM.

This study also provides intriguing insights into the real-world use and efficacy of 2L therapy. Historically, patients with bladder cancer have had a high rate of attrition across lines of treatment. In a recent study using Flatiron Health EMR data, Thomas et al. showed that only 37% of patients who commenced 1L treatment received 2L therapy, with only 12% reaching 3L [28]. There are hints in our study that this may be changing. We observed that nearly half of patients reached 2L treatment. Of the 96 patients (45%) who received 2L treatment post-avelumab 1LM, 53 (55%) received EV monotherapy. Outcomes from the start of 2L EV were comparable to those seen in the EV-301 (NCT03474107) trial despite a slightly shorter median treatment duration (4.2 vs. 5.0 months) [29]. Median rwOS from 2L EV initiation was 11.2 months (95% CI: 6.8—NE), compared with a median OS of 12.9 months (95% CI: 10.6–15.2) in EV-301, and rwPFS was 4.9 months (95% CI: 3.9–8.8) in this study, compared with a median PFS of 5.6 months (95% CI: 5.3–5.8) in EV-301. These outcomes from 2L EV initiation following avelumab 1LM are aligned with similar real-world studies in the US, including UNITE, which reported a median rwOS of 13.3 months (95% CI: 10.8—NE) and rwPFS of 7.0 months (95% CI: 5.8–13.3) [30], and from a study utilizing the Tempus EHR database that reported a median rwOS of 11.6 months (95% CI: 6.1—NE) and rwPFS of 6.6 months (95% CI: 4.1—NE) [22]. As reported in similar real-world studies [28], a high attrition across lines of therapy was reported, and outcomes in the 19% of patients (n = 40) with treatments beyond 2L remain to be explored.

The strengths of the current study include the heterogeneous population of patients drawn from a spotlight dataset capturing real-world patients with aUC who might not have been eligible for the JB100 trial, such as patients with an ECOG PS of ≥2. The oncologist-defined, rule-based LOTs and disease progression information contained in the deidentified database in this study provide insight into the real-world patient characteristics and treatment patterns (including eligibility for switch maintenance therapy) among early adopters of avelumab 1LM. The patient data contained in the Flatiron Health database are largely derived from community oncology practices, which is reflective of oncology care in the US where approximately 85% of patients receive care in a community oncology setting [31]. Additionally, survival outcomes were comparable with JB100 despite a shorter follow-up and treatment duration. Taken together, these factors illustrate the strengths of this study, providing insight into the real-world treatment patterns and outcomes in early adopters of avelumab 1LM, which remains a recommended regimen per NCCN Guidelines [4]. This study highlights the opportunity to further improve survival outcomes in patients by ensuring that the treatment strategy considering subsequent LOTs is supported by evidence-based recommendations, including the use of avelumab 1LM, which continues to be evaluated in ongoing, international, prospective, real-world studies including AVENUE in Europe [32] and SPADE in the Asia–Pacific region [33]. In consideration of the recent NCCN Guidelines change that recommends pembrolizumab plus EV as the preferred 1L therapy, careful sequencing of agents should be considered regarding factors including patient characteristics (age, comorbidities) and treatment burden (toxicity, dosing schedule, costs) [33]. As value-based oncology care is becoming increasingly important in the US, the lower costs of 1L PBC followed by avelumab 1LM compared with pembrolizumab plus EV should be taken into consideration [34].

Given that this analysis was based on retrospective, nonrandomized data, these results, while consistent with what is known from JB100 and other clinical trial data, should be interpreted with caution. As with all retrospective database studies, certain limitations apply, including the lack of patient randomization to treatment, prohibiting any causal inferences from being formed. The level of missing data for some variables in the database must be considered when interpreting the results of this study. Treatment sequences and discontinuation information are based on information in the patients’ EHRs. Tumor progression was based on radiologist assessment or physician notes, which is subject to the physician’s assessment. The complete care pathway is not captured in the Flatiron Health database, which does not contain inpatient data or complete medical history outside of the oncology EHR, which may have resulted in underreporting of treatments. In addition, cause of death data are not captured by the database, precluding a cancer-specific survival analysis. Clinical characteristics and lab values were frequently missing, which limits the interpretation of survival outcomes in this study. Namely, disease stage at initial diagnosis was not reported in 44% (n = 94) of patients, ECOG PS was not reported in 13% (n = 28) of patients, and PD-L1 status was missing in 70% (n = 150) of patients. Whereas disease stage at diagnosis was missing in 44% (n = 94) of patients, and 46% (n = 99) of patients were diagnosed with stage IV disease. Although this study included some patients with EGOG PS ≥ 2 (n = 21, 9.8%) who would not have been eligible for enrolment in JB100, most patients had an ECOG of 0 or 1, similar to JB100 [5]. Of note, while the JB100 trial included survival outcomes by PD-L1 status, a post hoc analysis reported that no significant interaction between treatment and PD-L1 status was observed for OS, supporting the use of avelumab 1LM, regardless of PD-L1 status [35]. Additionally, a recorded response to treatment was not reported in 20% (n = 43) of patients that received avelumab 1LM, and less is known about the determination of these responses, which was not verified by the same blinded centralized review process performed in clinical trials. The results reported in this study are based on data collected from centers included in the Flatiron EHR network and may not reflect practice patterns observed in other cancer treatment institutions or patient populations. Finally, the study period represents a limitation, capturing patients who received avelumab 1LM following US Food and Drug Administration approval in July 2020, through the end of the study period on December 31, 2022. As such, assessments were limited to approved treatments during that time, and treatments approved and incorporated into clinical guidelines in the time since the study period end have not been captured. An analysis of more recent data might reflect the use of therapies not available during the current study’s time period and also reflect changes in NCCN Guidelines.

## 5. Conclusions

This retrospective observational study of 214 patients with aUC treated with avelumab 1LM in the Flatiron Health network provides insights into the treatment patterns, sequencing, and clinical outcomes in real-world clinical practice in the post-avelumab 1LM approval era. This study sets an important baseline for treatment patterns, sequencing, and clinical outcomes amid a rapidly evolving US treatment landscape. Clinical outcomes including survival results are highly consistent with the JB100 clinical trial of avelumab 1LM vs. best supportive care alone in la/mUC that demonstrated improved outcomes, including in clinically relevant subgroups [6]. The body of evidence is strengthened by several US and European real-world studies and continues to grow with ongoing studies in Europe and the Asia–Pacific region, supporting avelumab 1LM efficacy in heterogenous patients treated in routine clinical practice. The results from this study support the continued use of avelumab 1LM following 1L PBC as a recommended therapy per NCCN Guidelines and other international treatment guidelines including the European Society for Medical Oncology [36] and the European Association of Urology [37]. Despite the limitations of retrospective analyses, this study provides insights into the sequencing and effectiveness of 2L EV among patients with la/mUC who are treated with avelumab 1LM after disease control with 1L PBC. Appropriate selection and sequence of therapies may optimize the proportion of patients who will remain well enough to continue therapy if and when there is disease progression during or after 1L therapy. As novel treatments for la/mUC, including pembrolizumab plus EV, are approved and incorporated into clinical practice, further research is needed to understand optimal treatment sequencing, thereby maximizing clinical outcomes in the real-world setting.

## Figures and Tables

**Table 1:** Baseline demographics and clinical characteristics.

Characteristic	Avelumab 1LM (n = 214)
**Age at la/mUC diagnosis, years**	
Mean (Std Dev)	69.0 (9.2)
Median (Q1, Q3)	70.0 (64.0, 76.0)
**Year of index date, n (%)**	
2020	43 (20.1)
2021	75 (35.0)
2022	96 (44.9)
**Sex, n (%)**	
Male	164 (76.6)
Female	50 (23.4)
**Race, n (%)**	
White	142 (66.4)
Other	31 (14.5)
Black or African American	6 (2.8)
Asian	2 (0.9)
Unknown	33 (15.4)
**Region of residence, n (%)**	
South	99 (46.3)
Northeast	37 (17.3)
Midwest	30 (14.0)
West	23 (10.7)
Other	1 (0.5)
Unknown	24 (11.2)
**Practice type, n (%)**	
Community	191 (89.3)
Academic	18 (8.4)
Both	5 (2.3)
**Site of disease, n (%)**	
Bladder	158 (73.8)
Renal pelvis	30 (14.0)
Ureter	24 (11.2)
Urethra	2 (0.9)
**Disease grade, n (%)**	
High grade (G2/G3/G4)	174 (81.3)
Low grade (G1)	12 (5.6)
Unknown/not documented	28 (13.1)
**Stage at initial diagnosis, n (%)**	
0	2 (0.9)
I	3 (1.4)
II	9 (4.2)
III	7 (3.3)
IV	99 (46.3)
Unknown/not documented	94 (43.9)
**ECOG PS at la/mUC diagnosis date, n (%)**	
0	69 (32.2)
1	96 (44.9)
2+	21 (9.8)
Unknown/not documented	28 (13.1)
**Body mass index, kg/m^2^, n (%)**	
Underweight (<18.5)	9 (4.2)
Normal (18.5–24.9)	69 (32.2)
Overweight (25–29.9)	59 (27.6)
Obese (≥30)	60 (28.0)
Unknown	17 (7.9)
**Sites of metastases, n (%)**	
Distant lymph node	122 (57.0)
Bone	63 (29.4)
Lung	58 (27.1)
Liver	37 (17.3)
Soft tissue	21 (9.8)
Peritoneum	8 (3.7)
Other	6 (2.8)
Pleura	5 (2.3)
Adrenal	5 (2.3)
Brain	2 (0.9)
Skin	1 (0.5)
Kidney	1 (0.5)
**PD-L1 status, n (%)**	
Positive	25 (11.7)
Negative	39 (18.2)
Unknown/not documented	150 (70.1)
**Follow-up time, months**	
Median (Q1, Q3)	8.7 (4.5, 15.7)
**1L carboplatin-based regimens (n = 99, 46.3%), n (%)**	
Carboplatin plus gemcitabine	98 (99.0)
Carboplatin plus paclitaxel	1 (1.0)
**1L cisplatin-based regimens (n = 115, 53.7%), n (%)**	
Cisplatin plus gemcitabine	101 (87.8)
MVAC	8 (7.0)
Cisplatin monotherapy	2 (1.7)
Cisplatin, bicalutamide, gemcitabine, and leuprolide	1 (0.9)
Cisplatin, gemcitabine, and hydroxyurea	1 (0.9)
Cisplatin plus paclitaxel	1 (0.9)
MVAC plus leuprolide	1 (0.9)
Best response to 1L PBC *, n (%)	
rwCR	14 (6.9)
rwPR	159 (78.7)
rwSD	29 (14.4)

Due to rounding, percentages may not add up to 100%. Abbreviations: 1L, first line; 1LM, first-line maintenance; ECOG PS, Eastern Cooperative Oncology Group performance status; G, grade; la/mUC, locally advanced/metastatic urothelial carcinoma; MVAC, methotrexate, vinblastine sulfate, doxorubicin, and cisplatin; PBC, platinum-based chemotherapy; Q1, first quartile; Q3, third quartile; rwCR, real-world complete response; rwPR, real-world partial response; rwSD, real-world stable disease; Std Dev, standard deviation. * 202 patients had a recorded response to 1L PBC.

**Table 2:** Clinical outcomes associated with patients treated with avelumab 1LM.

**Patients Treated with Avelumab 1LM (n = 214)**	
**Duration (months) of avelumab 1LM treatment, median (Q1, Q3)**	3.9 (1.9, 7.2)
**Median follow-up, months (Q1, Q3)**	8.7 (4.5, 15.7)
Best response to 1LM *, n (%)	
rwCR	16 (9.4)
rwPR	44 (25.7)
rwSD	44 (25.7)
**ORR, % (95% CI)**	35.1 (28.0–42.7)
**Outcomes from 1LM initiation, median (95% CI), months**	
rwOS	23.8 (18.2—NE)
rwPFS	5.1 (4.1–7.0)
TTD	4.9 (4.2–6.5)
TTNT	7.0 (5.6–8.6)
**Patients Treated with Subsequent 2L EV (n = 53)**	
**Duration (months) of 2L EV treatment, median (Q1, Q3)**	4.2 (1.6, 6.3)
**Outcomes from 2L EV initiation, median (95% CI), months**	
rwOS	11.2 (6.8—NE)
rwPFS	4.9 (3.9–8.8)
TTD	4.7 (4.2–8.5)
TTNT	5.8 (5.2–9.5)

Abbreviations: 1LM, first-line maintenance; 2L, second line; EV, enfortumab vedotin; NE, not estimable; ORR, objective response rate; Q1, first quartile; Q3, third quartile; rwCR, real-world complete response; rwOS, real-world overall survival; rwPFS, real-world progression-free survival; rwPR, real-world partial response; rwSD, real-world stable disease; TTD, time to treatment discontinuation; TTNT, time to next treatment. * 171 patients had a recorded response to avelumab 1LM.

## Data Availability

The data supporting the findings of this study are available from Flatiron Health. Requests for data sharing by license or by permission for the specific purpose of replicating results in this manuscript can be submitted to publicationsdataaccess@flatiron.com.
